# Tarrant County Medical Society

**Published:** 1914-12

**Authors:** 


TARRANT COUNTY MEDICAL SOCIETY.—At a well attended meeting of the Tarrant County Medical Society in the auditorium of the Medical College, on November 6th, Dr. J. A. Gracey read a paper on "Seeing Things at the Bedside." Dr. Gracey reviewed his work as a practitioner, which extends over a period of thirty years. It was freely discussed by the doctors present. Dr. I. L. Van Zandt and Dr. R. W. Moore reported a number of interesting cases, which were discussed.

Attention of the law enforcement committee was called to the fact that several physicians are practicing in Fort Worth without licenses.

Two new members were elected—Dr. Charles H. McCollum and Dr. Bessie Vandeventer. The secretary reported a number of new applications.

In the absence of Dr. S. A. Woodward, Dr. Gracey presided.

---

DENTON COUNTY MEDICOS met at Dr. M. C. McBride's office Tuesday afternoon, November 3d, with Drs. Buster and Harris, of Pilot Point; Roark, of Roanoke, and Rice, of Sanger, the out-of-town visitors.

Dr. Crumb of the Parke, Davis Drug Co. made a short talk on the serum treatment of disease, and there were other discussions.

---

MEMBERS of the McLennan County Medical Society met in Waco November 3d. Three interesting addresses were delivered. Dr. G. A. Kindley, of Temple, talked on "Acute Intestinal Intoxication," and Dr. J. H. Wysong, of Hico, on "Auto-intoxication." Dr. H. C. Black also made an address, the subject being selected by him.

A committee report was received on the appointment of a district course. There were several other important committee reports.

The Williamson County Medical Society met in regular session in the county court room Wednesday, October 14th, Dr. T. M. Harrell, President, in the chair.

Minutes of previous meeting read and approved.

The following members were present: O. B. Atkinson, Florence; O. G. Bundy, Hutto; C. W. Thornton, W. G. Pettus, B. Nowlin, E. M. Thomas, G. W. Foster, S. S. Martin, G. K. Talley, G. E. Henschen and E. M. Wood, of Georgetown; W. G. Webber and T. M. Harrell, Round Rock; W. L. Helms, Jonah; C. C. Foster, W. A. Winn and D. M. Cooke, Granger; E. M. Stormberg and G. A. Wedemeyer, Taylor; J. F. Taylor, Briggs; C. L. Simmons, W. D. Fowler and J. H. Vaughan, Liberty Hill; G. L. Robertson, Leander; H. Keuhn, Walburg.

Visitors: J. C. Thomas, Taylor; Z. T. Scott and E. G. Mathis, Austin, and C. S. Venable, San Antonio.

Dr. W. L. Crosthwait, of Waco, notified the Society that it would be impossible to be present, but sent his paper to the secretary, and was read by secretary and was generally discussed and very much appreciated.

Dr. C. S. Venable, of San Antonio, discussed Pott's disease, with presentation of case. This was an especially interesting discussion and was enjoyed by all present. Dr. J. C. Thomas, of Taylor, opened the discussion.

E. M. Wood, Secretary.

